# Supplementary figures and images for: A model of antibiotic resistance genes accumulation through lifetime exposure from food intake and antibiotic treatment
Source: PLoS One. 2023 Aug 17;18(8):e0289941. doi: 10.1371/journal.pone.0289941 (PMC10434901; doi:10.1371/journal.pone.0289941)

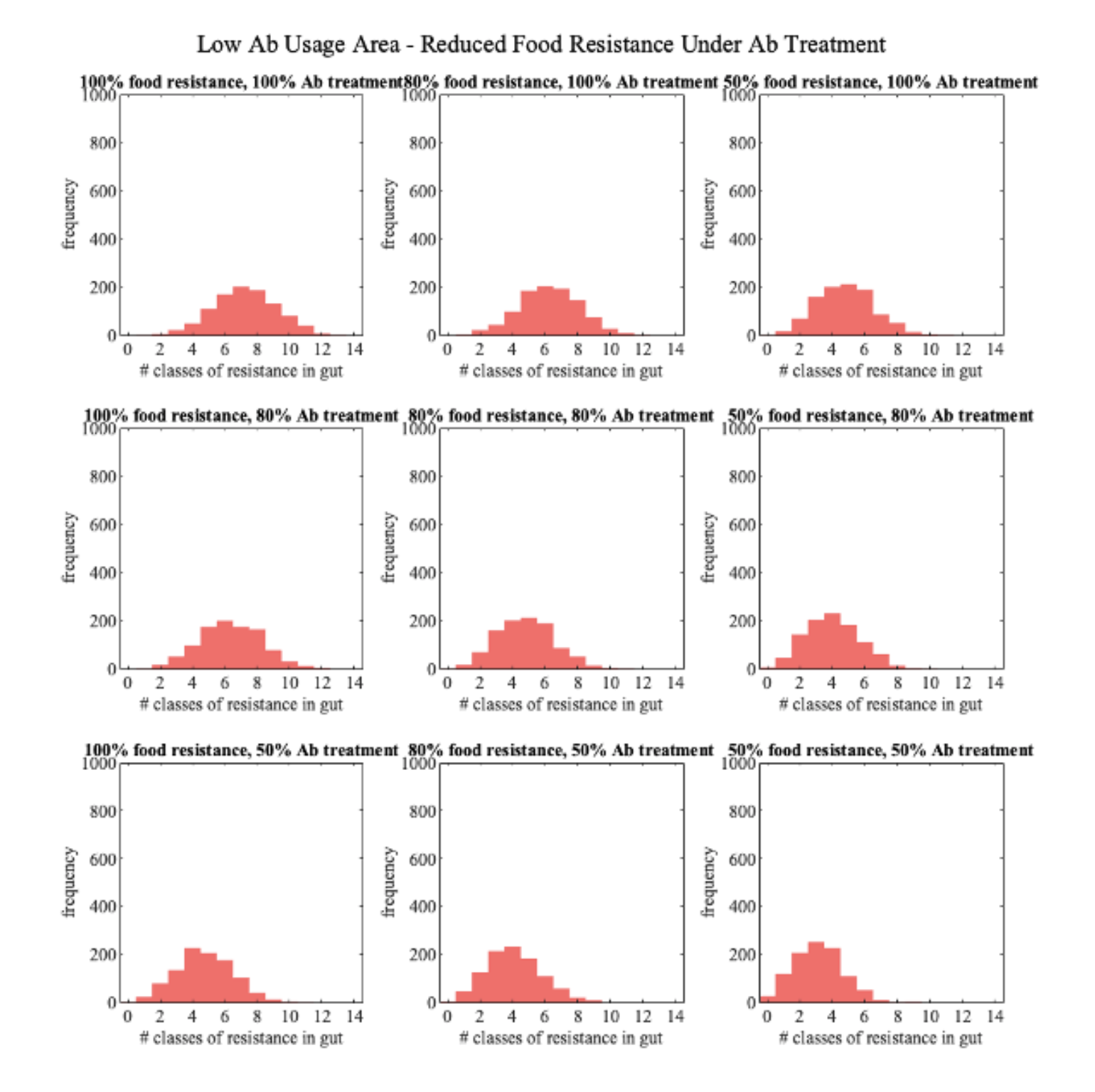

Supplement: S1 Fig — (TIF) [file pone.0289941.s001.tif]

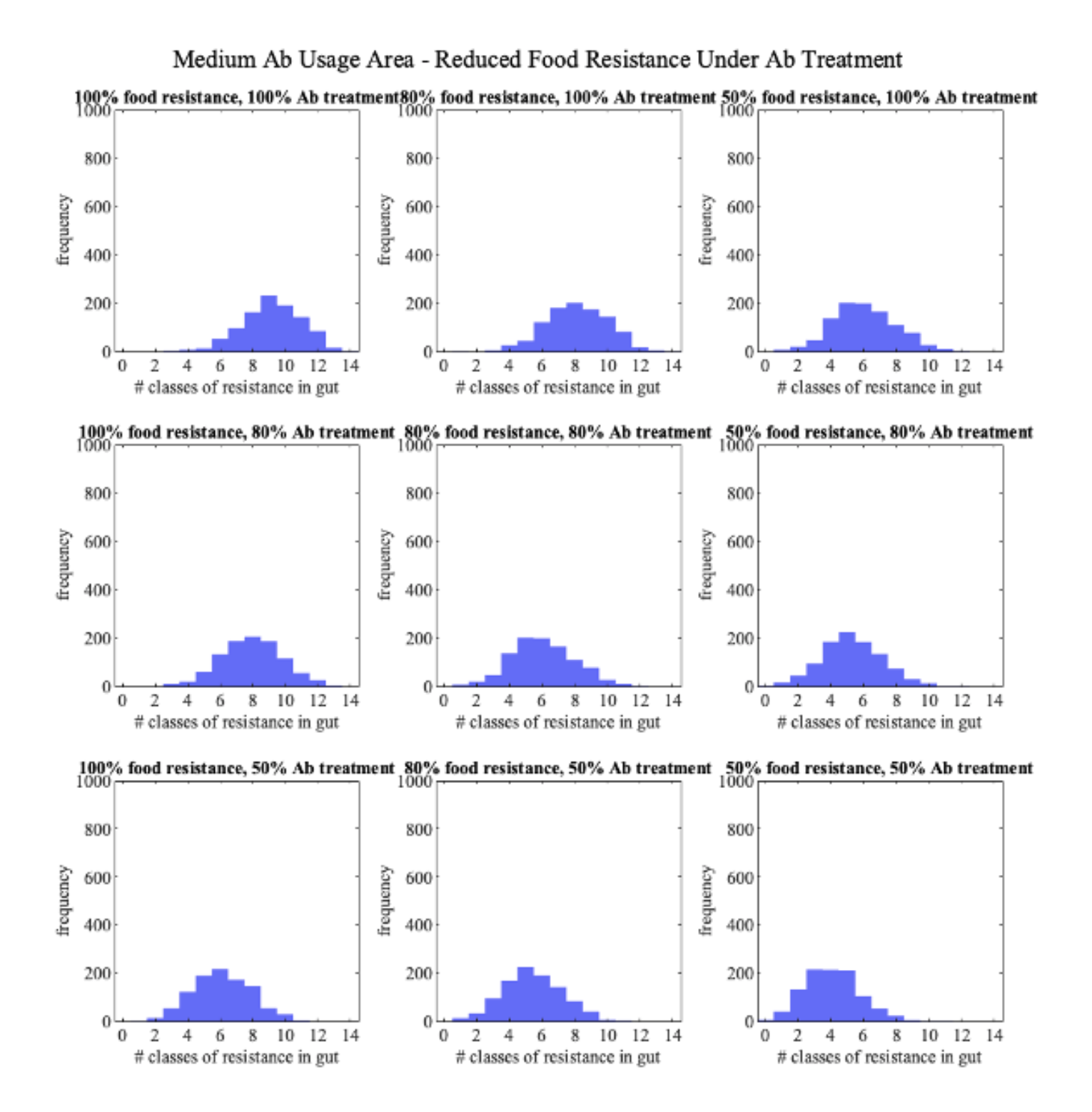

Supplement: S2 Fig — (TIF) [file pone.0289941.s002.tif]
