# Supplementary material for: A model of antibiotic resistance genes accumulation through lifetime exposure from food intake and antibiotic treatment
Source: PLoS One. 2023 Aug 17;18(8):e0289941. doi: 10.1371/journal.pone.0289941 (PMC10434901; doi:10.1371/journal.pone.0289941)
Supplement: Attachment — Submitted filename: Food_Model_PLoS-Reviewer.pdf [file pone.0289941.s004.pdf]

A model of antibiotic resistance genes accumulation through lifetime exposure from food intake and antibiotic treatment

Henry Todman[1], Sankalp Arya[1¤a], Michelle Baker[1¤b], Dov J Stekel[1,2*],

1 School of Biosciences, University of Nottingham, Sutton Bonington Campus, Loughborough, Nottingham, UK
2 Department of Mathematics and Applied Mathematics, University of Johannesburg, Rossmore 2029, South Africa

¤a Current Affiliation: Department of Mathematics, North Carolina State University, Raleigh, NC 27695-8205, USA ¤b Current Affiliation: School of Veterinary Medicine and Science, University of Nottingham, Sutton Bonington Campus, Loughborough, Nottingham, UK
* dov.stekel@nottingham.ac.uk

Abstract

Antimicrobial resistant bacterial infections represent one of the most serious contemporary global healthcare crises . Acquisition and spread of resistant infections can occur through community, hospitals, food, water or endogenous bacteria. Global efforts to reduce resistance have typically focussed on antibiotic use, hygiene and sanitation and drug discovery. However, resistance in endogenous infections, e.g. many urinary tract infections, can result from life-long acquisition and persistence of resistance genes in commensal microbial flora of individual patients, which is not normally considered. Here, using individual based Monte Carlo models calibrated using antibiotic use data and human gut resistomes, we show that the long-term increase in resistance in human gut microbiomes can be substantially lowered by reducing exposure to resistance genes found food and water, alongside reduced medical antibiotic use. Reduced dietary exposure is especially important during patient antibiotic treatment because of increased selection for resistance gene retention; inappropriate use of antibiotics can be directly harmful to the patient being treated for the same reason. We conclude that a holistic approach to antimicrobial resistance that additionally incorporates food production and dietary considerations will be more effective in reducing resistant infections than a purely medical-based approach.

Introduction

The human gut is a diverse and dynamic environment playing host to a wide variety of bacteria, viruses, archaea and eukaryotes. Genomic studies have shown that the human gut microbiota contains more than 300 species of bacteria [1]. These commensal enteric bacteria are thought to be largely harmless and play an important role in maintaining the health of the host through various mechanisms: i.e. protection against colonisation by pathogens through competitive exclusion or production of antimicrobial chemicals [2–4], maintaining the host immune system [3, 5], and extracting energy and nutrients from food [3]). However, at lower levels there are pathogenic bacteria endogenous to the gut (in particular *Enterobacteriaceae* and *Enterococcaceae*) [6].

In addition to playing host to a diverse collection of bacteria and other micro-organism, the human gut represents a reservoir of antimicrobial resistance (AMR), with antimicrobial resistance gene (ARG) abundance known to be correlated at a population scale with antibiotic use [7]. Enteric bacteria hold ARGs either on their chromosomes or on mobile genetic elements; it is thought that these ARGs predominantly reside within non-pathogenic unclassified species [8].

Antimicrobial resistances in the gut may become established after ingestion of contaminated food. Many studies have shown ARGs to be present in a range of high-risk food products: raw and cooked meats [9–13], fermented milk products [14–16], fermented meat products [17,18] and vegetable products [19–21]. The ready-to-eat food market is particularly problematic due to a lack of cooking and washing before consumption [22–25].

Several authors have identified systematic differences in ARG levels between individuals resident in different countries [7,26,27]. One source of these differences is likely to result from differing levels of availability of antibiotic treatments. Governmental approaches to antibiotic availability are diverse and defined daily doses per inhabitant can vary widely [7,28,29].

Ageing individuals are at higher risk of AMR infections because of increased exposure to ARGs, increased lifetime exposure to antibiotics and increased vulnerability to infection with age. The number of ARGs within an individual's intestinal tract is correlated with age [30,31]. Lu *et al.* [30] showed that the number of resistances from faecal samples of four different age groups were positively correlated with increasing age. Further, cluster analysis suggested that resistances were being acquired and accumulated over time rather than being transient. Other research has also supported this view. Ghosh *et al.* [8] profiled resistance genes of 275 gut flora samples sourced from multiple countries and found increasing ARG diversity with age. Antimicrobial resistant bacterial infections in older age can often result from endogenous bacteria moving from the intestinal tract to other areas of the body, for example the urinary tract [32,33].

In this work, we bring together these aspects of ARG establishment using a probabilistic mathematical model of the accumulation of antimicrobial resistance over an individual lifetime. We consider four key model parameters: antibiotic use, ARG ingestion, and the probabilities of an ARG becoming established in the presence and absence of concurrent antibiotic use. Taking reported demographic data, we show how accumulation of resistances are likely to vary by country depending on antibiotic availability and use. We show that alterations to food intake during antibiotic treatment can reduce the overall antibiotic resistance level in an individual.

Methods

We have defined a probabilistic model to define the acquisition of ARGs in the enteric system of individuals (figure 1), which we have evaluated using Monte Carlo simulations. We consider the acquisition of resistance genes to 14 different classes of antibiotics. In order to reduce model complexity, we consider resistance to individual classes of antibiotics (e.g. beta lactams, carbapenems, cepholosporins, aminoglycosides, etc), rather than specific antibiotics. This is purposefully simplified view of resistance to different antibiotics: there is significant variation in resistance genes of antibiotics within the same class and indeed variation between ARGs conferring resistance to the same antibiotic (e.g. there are over 40 different genes divided into 11 different classes of action which encode resistance to tetracycline [34]).

For each antibiotic class, we consider the probability that an individual is exposed to resistance genes through ingestion of food, and the probability of these resistance genes becoming fixed in the individual's enteric bacterial communities. The probability of

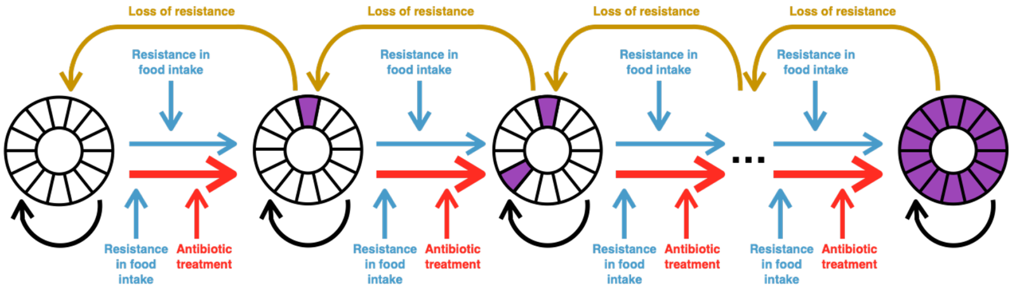

Fig 1. Schematic diagram of the lifetime food model showing key model interactions.

resistance becoming established in the gut microbiome is dependant on whether the individual may be undergoing antibiotic treatment (of the same class as the resistance genes), as the presence of antibiotic treatment provides selective pressure for these resistance genes.

For each class of antibiotic, we consider the probability of exposure to resistance genes for that antibiotic via the food chain, as well as the probability of resistance becoming established in the gut microbiome in the presence and absence of selective pressures from concurrent antibiotic treatment. For simplicity, once acquired and established, we assume that the resistance will persist in the commensal flora of an individual throughout their lifetime; this is in accordance with metagenomics studies [30].

Throughout our analysis we considered three different levels of antibiotic use, which reflect different national levels of antibiotic availability. We ran different simulations of this model, with each scenario for 1000 individuals. We have based the parameter values for the probability of antibiotic use in areas with low and medium antibiotic usage from drug utilization figures for European countries [26], and then estimated high antibiotic use areas parameter values based on this.

We simulate individual lifetimes in the Monte Carlo model with time steps of one week. The probability of an individual being exposed to resistance via food intake each week ($P_{\text{FoodRes.}}$) is a random variable with a uniform distribution. Once exposed to resistance, there is a probability that this resistance shall establish in the microbial flora in the gut of the individual (P_{Fix}). Each week, there is an independent probability that the individual may undergo antibiotic treatment ($P_{\text{Ab. Treat.}}$). As the use of antibiotics can exert selective pressures on resistant bacterial populations, we assign a greater probability of establishment of ARGs in the presence of antibiotic treatment ($P_{\text{Ab. Fix.}}$). The probability that the individual acquires new class of resistance in any given week is given by the transition probability (1).

$$\mu_i = P_{\text{food res.}}(A_i)\left((1 - P_{\text{Ab. treat}}(A_i))\, P_{Fix}(A_i) + P_{\text{Ab. treat}}(A_i) P_{Ab.Fix}(A_i)\right) \qquad (1)$$

The parameters used for each scenario are given in the table 1. At each time step in the model, we sample the probability of resistance in food intake from a continuous uniform distribution $U(0, 0.5)$: this distribution is chosen to reflect intake of a varied diet from a variety of food sources, with the upper bound based on observed frequencies of resistance in E. coli isolates from food products [35, 36]. Then we estimate probabilities of resistance becoming established for this model based on metagenomic data for human gut microbiota in areas of different levels of antibiotic use [30].

We conducted a local sensitivity analysis for each of the model parameters. For each parameter, we take 1000 parameter values sampled from the feasible parameter space

Table 1. The standard parameters used when simulating the lifetime resistance model.

Parameter	Parameter Name	Parameter Values	Parameter Range
N	Number of antibiotic classes considered	14	-
n	Number of individuals simulated	1000	-
$P_{\text{Ab. Treat}}(A_i)$	Probability of an individual receiving antibiotic treatment for antibiotic class A_i	1.4×10^{-2} (low Ab usage) 2.1×10^{-2}(medium Ab usage) 5.0×10^{-2} (high Ab usage)	0 - 1
β_{food}	Upper bound for uniform distribution of $P_{\text{food res.}}(A_i)$	0.5	0 - 1
$P_{\text{food res.}}(A_i)$	Probability of resistance genes for antibiotic class A_i being present in an individual's food intake	$P_{\text{food res.}}(A_i) \sim \mathcal{U}(0, \beta_{\text{food}})$	0 - β_{food}
$P_{\text{Fix}}(A_i)$	Probability of resistance genes becoming established in an individuals resistome in absence of antibiotics	1.0×10^{-4}	0 - 1
$P_{\text{Ab. Fix}}(A_i)$	Probability of resistance genes for antibiotic class A_i becoming established in an individuals resistome in presence of antibiotics	5.0×10^{-2}	0 - 1
$P_{\text{Loss}}(A_i)$	Probability of resistance genes for antibiotic class A_i being lost	1.0×10^{-6}	0 - 1

(table 1) and calculate the mean resistance load for each. We also tested sensitivity to gene loss, by constructucting an alternative model which includes the possibility of acquired resistance genes to be lost. In order to simulate the possible loss of ARGs from the resistome through wash out, at the end of each time step in the Markov chain model there is a possibility that an acquired resistance, A_i, is lost with probability $P_{\text{Loss}}(A_i)$.

MATLAB R2020b was used to run time course simulations of the lifetime resistance model and to perform a local sensitivity parameter analysis of the model.

Results & Discussion

Simulation of 1000 individuals in each of the 3 antibiotic usage scenaios (low, medium and high) for the standard parameter set (table 1) shows that higher antibiotic use increases ARG acquisition over time by commensal bacteria (figure 2).

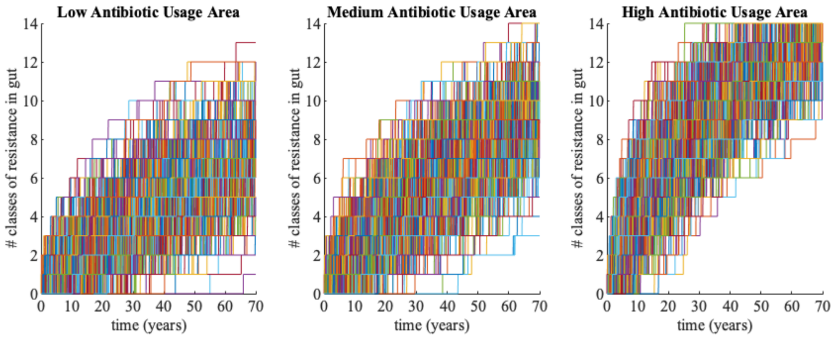

(a) Timecourse of lifetime resitance model

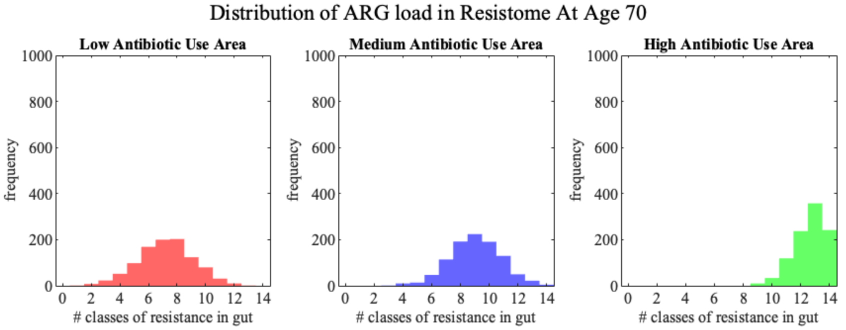

(b) Histogram showing the distribution of ARG load in individuals' resistomes

Fig 2. (a) Time course simulation of Lifetime resistance model for low, medium and high antibiotic use countries. In each of the three antibiotic use scenarios (low, medium and high), we have run the lifetime resistance model using the standard parameter set (given in table 1) for 1000 individuals. Each line represents an individual simulated in the lifetime resistance model. We can clearly see that individuals acquire more ARGs more quickly in areas of higher antibiotic usage. **(b) Histogram showing the distribution of ARG load in individual's resistomes by age 70 for the lifetime resistance model.** These histograms show the distribution of the number of resistance classes at the end of the time course simulations of 1000 individuals shown in (a) (i.e. at age 70). The mean and standard deviation, (μ, σ), for low, medium and high antimicrobial use areas are (7.2120, 1.9475), (9.0410, 1.7946) and (12.6250, 1.1321) respectively.

The average resistance load in individuals, at 70 years of age, from medium and high antibiotic use areas is 24.38% and 71.40% higher, respectively, than low antibiotic use areas. Thus our model concurs with many researchers and organisations including WHO [37], in advocating a reduction in antibiotic usage as a way to control the spread of antibiotic resistance, among other strategies. This strategy reduces opportunities for resistance genes to be the selected for in the gut over the lifetime of the individual.

In order to frame analysis of potential intervention on long term accumulation of resistance, we carried out local sensitivity analyses of the key parameters from the

model (figure 3). The accumulation of resistance is most sensitive to the probability of antibiotic treatment, and the probability of resistance fixing during antibiotic use. This effect is greatest in low and medium use countries. This led us to investigate impact of those factors in greater detail.

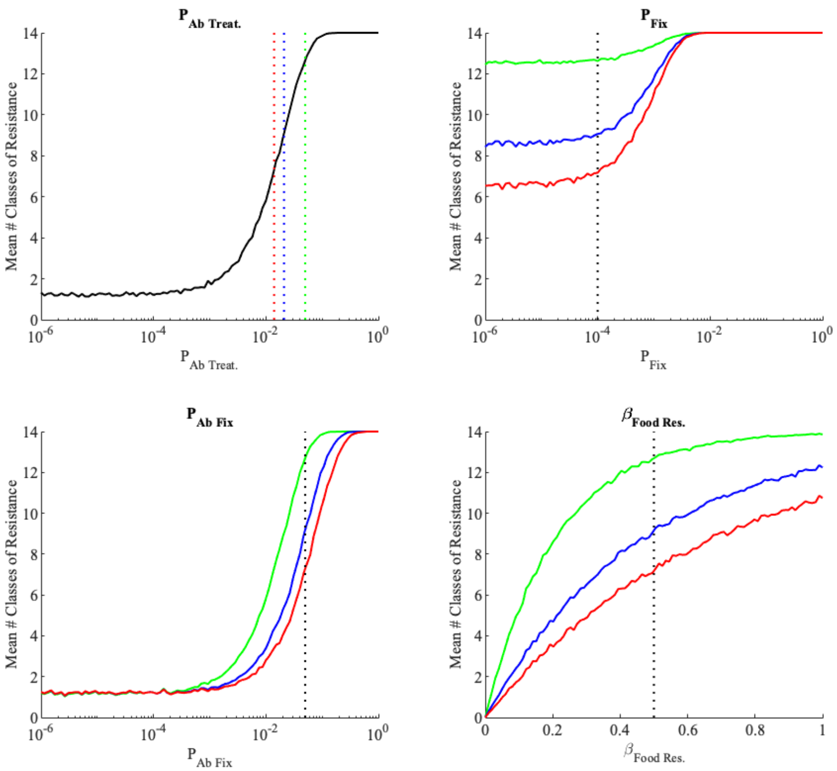

Fig 3. Local sensitivity analysis of the lifetime resistance model parameters. We vary the model parameters ($P_{\text{Ab. Treat.}}$, P_{Fix}, $P_{\text{Ab. Fix}}$, and β_{food}) across the possible parameter space (given in table 1) and then calculated the mean ARG load at age 70 of 1000 individuals for each of the different parameter values. For $P_{\text{Ab. Treat.}}$, the black line shows the mean resistance load as the probability of undergoing antibiotic treatment is varied across the parameter space, and the dashed red, blue and green lines indicate the parameter values used for $P_{\text{Ab. Treat.}}$in the low, medium and high antibiotic use areas respectively. For the local sensitivity analyses of P_{Fix}, $P_{\text{Ab. Fix}}$, and $\beta_{\text{food res.}}$, the red, blue and green lines represent the average resistance load as the parameter of interest is varied for low, medium and high antibiotic use areas respectively. The dashed black line in these subplots represents the values used for these parameters in the model simulations.

The number of resistance genes acquired by an individual is dependent on the use of antibiotics over the individual's lifetime and can be meaningfully reduced by a reduction in an individual's intake of ARGs through food.

We further explored the impact of a reduction in Ab usage by running simulations with a 20% and 50% reduction in the probability of an individual undergoing treatment in

any given week (figure 4). This analysis explores the practical effects of an Ab reduction policy in countries such as Denmark (low usage), Spain (medium usage) or China (high usage).

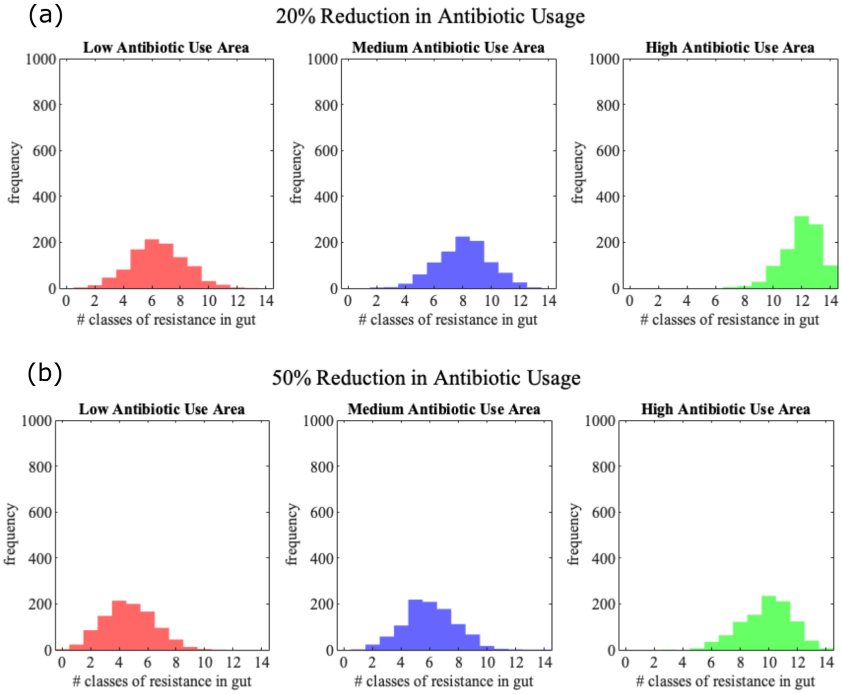

Fig 4. Histograms showing distribution of ARG load in individual by age 70 when there is (a) 20% and (b) 50% reduction in the probability of antibiotic treatment. The mean and standard deviation, $(\mu,\ \sigma)$, for the low antibiotic use area are $(6.4400,\ 1.9042)$ and $(4.6970,\ 1.7985)$ for the 20% and 50% reduction respectively. Similarly $(\mu,\ \sigma)$ for the medium and high antibiotic use areas is $(8.0660,\ 1.8604)$ and $(11.9670,\ 1.3243)$ respectively when antibiotic use is reduced by 20%, and $(6.0160,\ 1.8160)$ and $(9.8370,\ 1.7770)$ respectively when reduced by 50%.

Another avenue of control is the reduction of ARG intake through food and water. We considered two possibilities: an overall reduction of ARGs in food (and water) at all times; and a reduction only during antibiotic treatment, representing dietary change during such treatment, e.g. avoiding higher risk or raw foods. We considered two levels of reduction, 20% and 50% for both antibiotic usage and ARG levels in food. All scenarios were applied for low, medium and high antibiotic use countries. Figure 4 shows the effect of reducing the probability of undergoing antibiotic treatment alone and figure 5 the effect of reducing the probability of ARG in food alone. As expected, we observe that both these approaches result in a reduction in ARG acquisition over a lifetime, and reducing either ARG intake or antibiotic consumption by 50% gives a greater effect than reducing by 20%.

Reducing the antibiotic consumption in areas that have a higher rate of antibiotic treatment is on average more effective at reducing the ARG load: a 20% reduction in Ab usage yields an average 5.98% and 11.33% reduction in resistance load by age 70 in high and medium use areas respectively, while a 50% reduces the mean resistance load by 21.21% and 32.92%. Comparatively a reduction in ARG intake via food is more effective at reducing ARG acquisition in low antibiotic usage areas with a 20% and 50% reduction in ARG intake giving a 13.11% and 35.23% reduction in the mean resistance

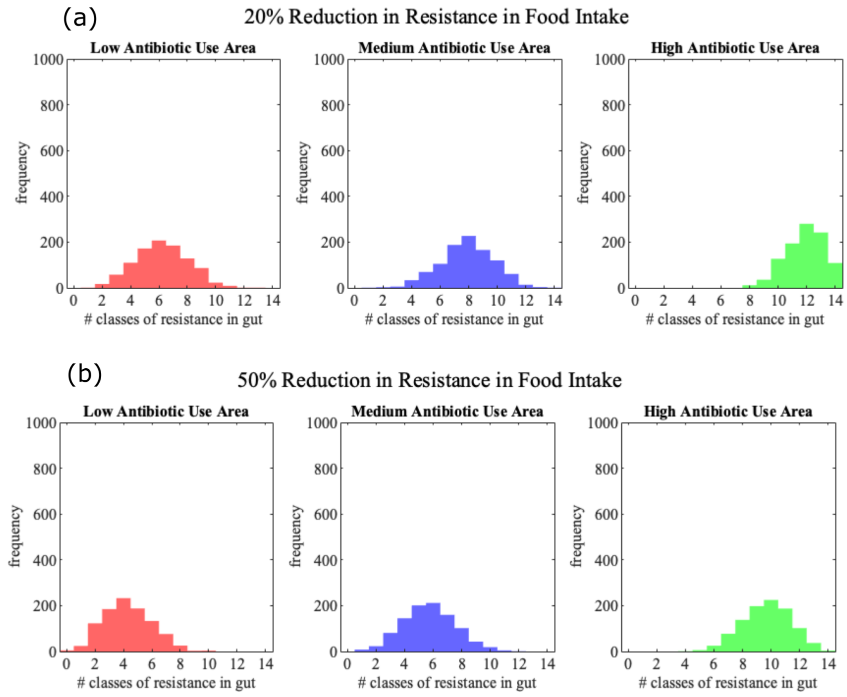

Fig 5. Histograms showing distribution of ARG load in individual by age 70 under a 20% and 50% reduction in resistance genes in an individual's food intake. Simulated using 1000 individuals in each of the case for each of the 3 Ab usage areas (low, medium & high). [Mean, Std, Max] for 20% food resistance reduction are [6.2150, 1.8842, 13], [7.8720, 1.9056, 14] and [11.8560, 1.7087, 14] in Low, Medium and High areas respectively. For 50% reduction [4.3020, 1.7099, 10], [5.7340, 1.8522, 12] and [9.677, 1.7087, 14] .

load.

Reducing ARG intake via food during periods of antibiotic treatment is particularly effective at limiting acquisition of resistance genes.

The greatest reduction in the number of resistance classes acquired by 70 years comes for a combined approach. Here for even a modest 20% decrease in both actions we see a reduction of between 12.63% and 24.02%, depending upon the original level antibiotic usage. For a 50% decrease in both, we observe the number of resistance classes acquired by 70 years can be reduced significantly (by between 46.35% and 56.52%) reducing the likelihood that endogenous bacterial infections in older age will be resistant to treatment. For the case of reduction only at times of antibiotic use, we saw a similar reduction in ARG load as for the reduced probability of ARG in food in general (Figure 6, S1 Fig & S2 Fig).

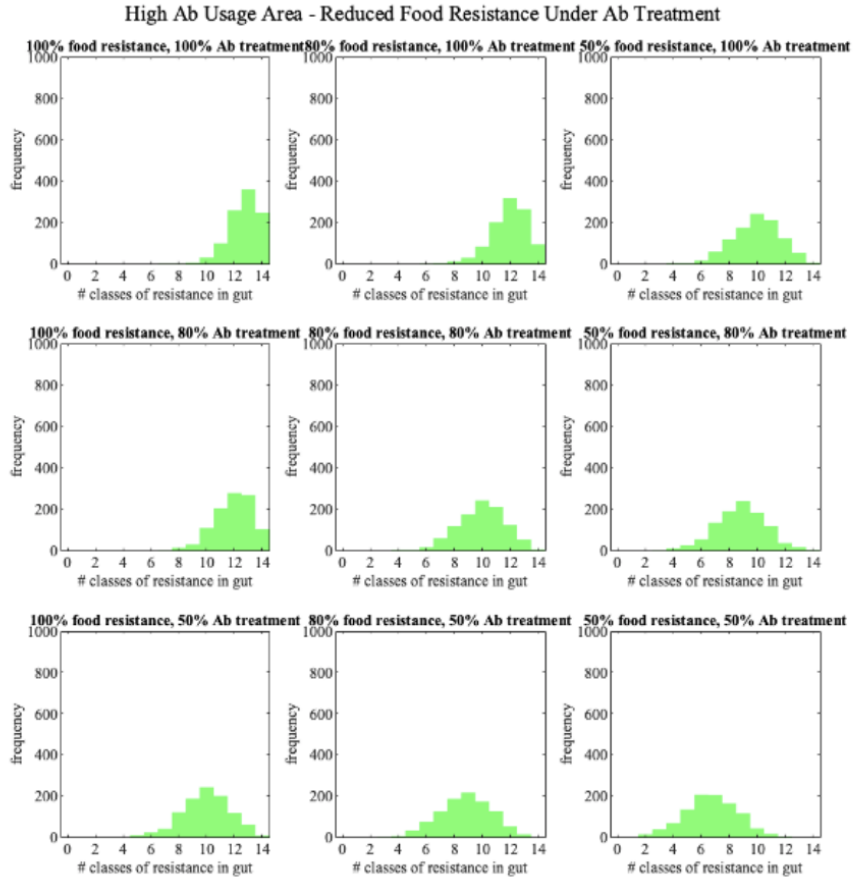

Fig 6. Distributions of ARG load in resistome for different levels of intervention strategies in high antibiotic usage area. Simulated using 1000 individuals in each intervention case.

Inclusion of ARG loss from resistome does not significantly impact lifetime resistance model simulations for realistic values of $P_{\mathbf{Loss}}$.

We adapted the lifetime model to include the possibility of resistance loss due to ARG washout or other factors. At the end of each time step in the markov chain model, there is a possibility that an acquired resistance, A_i, may be lost with probability $P_{\text{loss}}(A_i)$. We then simulated the lifetime model with ARG loss (figure 7(a)) for 1000 individuals in each of the three antibiotic usage areas using the parameter values given in table 1. A comparison of the results of the simulation with ARG loss, figure 7(a), and without ARG loss, figure 2(b), shows negligible differences between the models for each of the three antibiotic usage scenarios considered.

We then performed a local sensitivity analysis of the lifetime model with ARG loss to the parameter P_{loss} (figure 7(b)). Sensitivity analysis showed that the average resistance load was consistent with the standard lifetime model without ARG loss when the probability of resistance loss was less than 10^{-4}, while for P_{loss} greater than 10^{-4}, we see that the chance of ARG loss is high enough that it leads to a significant reduction on the average resistance load. However, it is important to note that this

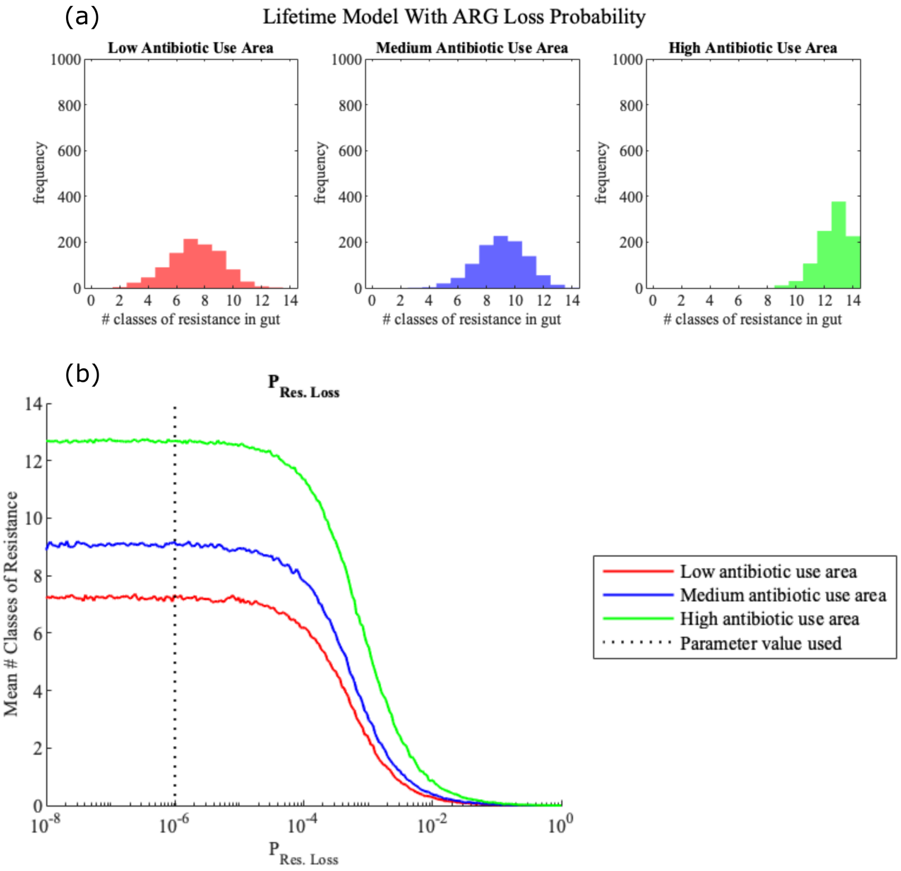

Fig 7. (a) Histogram showing the distribution of ARG load in individual's resistomes by age 70 for the lifetime resistance model including ARG washout. These histograms have been made by simulating the lifetime resistance model for 1000 individuals in each of the three antibiotic usage areas, where at each time step in the model there is a possibility that an individual may lose a resistance with probability $P_{\text{Loss}} = 1 \times 10^{-6}$. The mean and standard deviation, $(\mu,\ \sigma)$, for the low, medium and high antibiotic use areas are $(7.3330,\ 1.8841)$, $(9.0780,\ 1.7131)$ and $(12.6290,\ 1.1012)$ respectively.
(b) Local parameter sensitivity analysis of lifetime resistance model to the probability of resistance gene loss. We vary the value of the probability of an individual losing an acquired resistance, P_{Loss}, across the realistic parameter space (given in table 1) and then calculated the mean ARG load at age 70 of 1000 individuals for each of the different parameter values.

threshold probability of 10^{-4} is much higher than we would expect to see for the probability of ARG washout and the physically relevant parameter space for P_{loss} is expected to be $[10^{-7}, 10^{-5}]$.

Conclusion

We have shown that the long-term acquisition and retainment of genes providing resistance to different classes of antibiotics can be reduced by at three implementable

factors. First, the number of resistance genes acquired by and individual is dependent upon the use of antibiotics over an individual's lifetime. A conservative approach to antibiotic availability and dosing guidelines, as already implemented in many countries, and as advocated in much of literature on antibiotic resistance, would be a practical approach to reducing the long-term number of acquired resistances. Indeed, the converse is true, in that unnecessary antibiotic treatment can lead to long-term harm to the patient being treated, and could be considered unethical; this argument stands in contrast to the more standard argument that the risk over-use is primarily to patients other than the one being treated. Second, the number of acquired genes can be reduced even further if an individual's intake of resistance genes, carried on both pathogenic and non-pathogenic bacteria, is also reduced. This could be achieved by policy and practice changes in the food supply chain, including agriculture and post-harvest food production. Third, the reduction in intake of resistance genes is particularly effective during periods of antibiotic treatment where selective pressures increase the likelihood of the retainment of genes. We would suggest that dietary advice should be given to those undergoing antibiotic treatment to avoid products at higher risk of carrying ARGs (even on non-pathogens), as well as ensuring that all food consumed during treatment is fully cooked. The level of benefit to be gained from alterations in medical treatment and dietary changes is highly dependent upon the level of antibiotic use, which varies greatly between countries. Whilst our general model shows benefit at all prescribing levels, a differentiated model looking at region- and country-specific practices, as well as containing specific details of antibiotic classes and associated resistance genes, would be better able to be quantify the potential benefits of such changes.

Supporting information

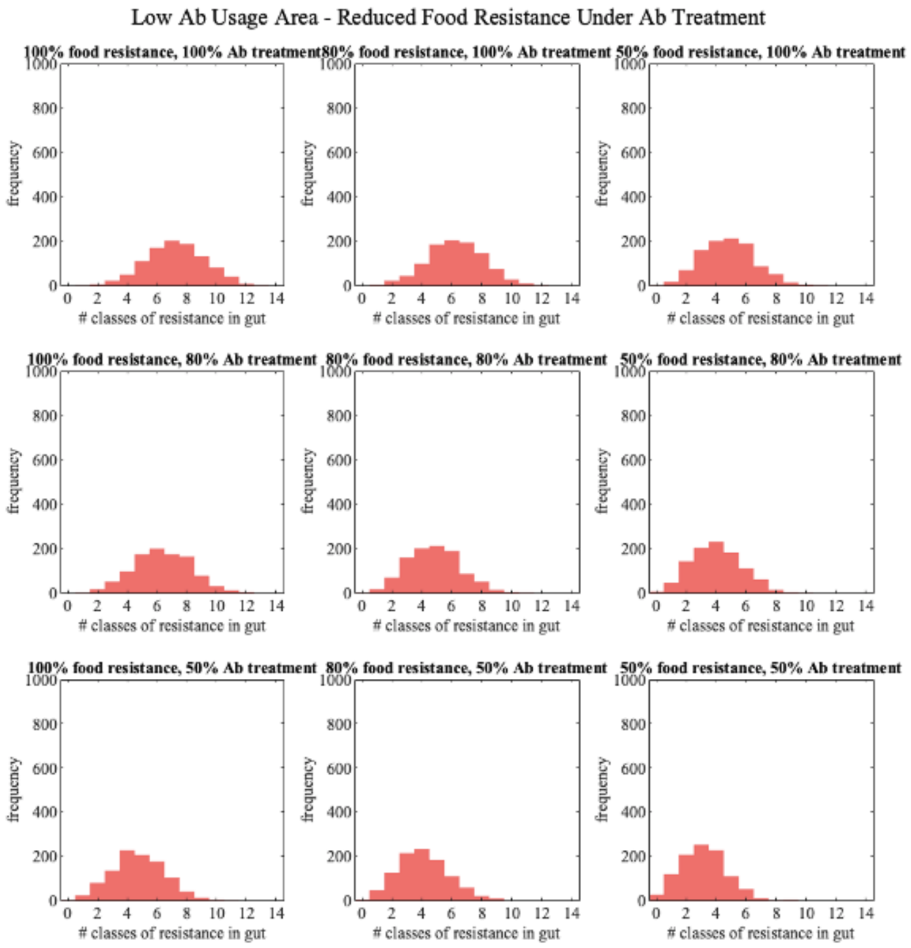

S1 Fig. Distributions of ARG load in resistome for different intervention strategies in low antibiotic usage area.

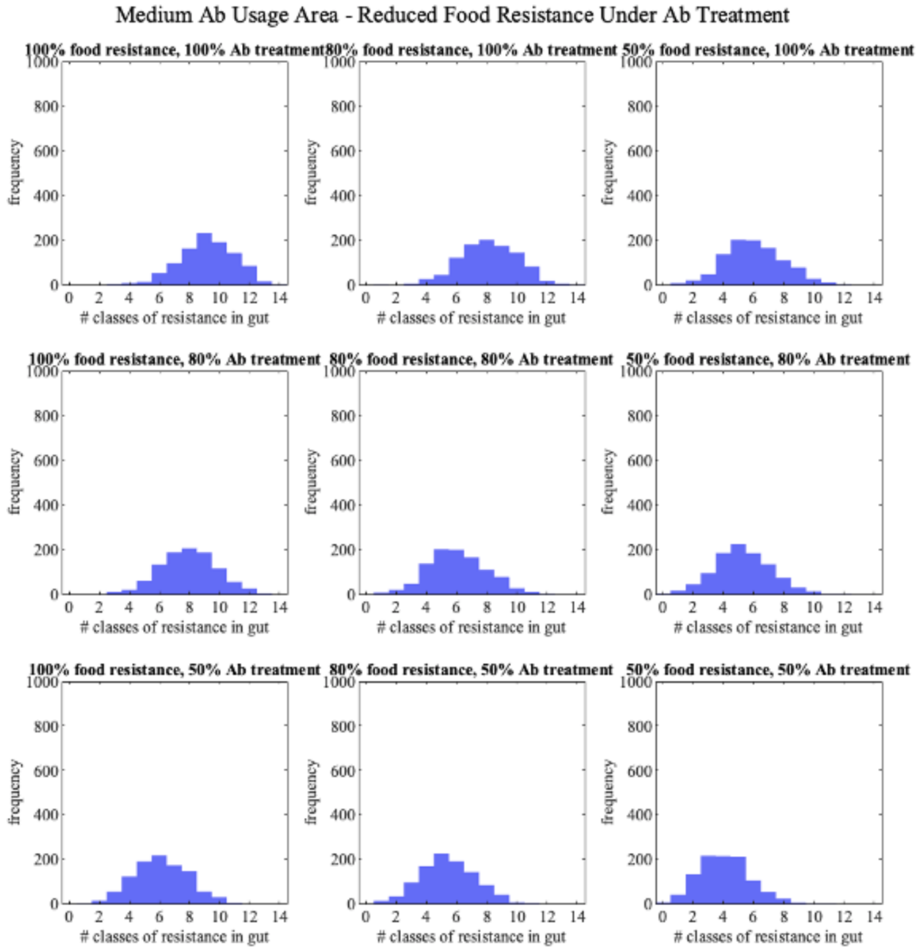

S2 Fig. Distributions of ARG load in resistome for different intervention strategies in medium antibiotic usage area. 209 210

References

1. Eckburg PB, Bik EM, Bernstein CN, Purdom E, Dethlefsen L, Sargent M, et al. Diversity of the human intestinal microbial flora. Science. 2005;308(5728):1635–1638. doi:https://doi.org/10.1126/science.1110591.

2. Kamada N, Seo SU, Chen GY, Núñez G. Role of the gut microbiota in immunity and inflammatory disease. Nat Rev Immunol. 2013;13(5):321–335. doi:10.1038/nri3430.

3. Wang B, Yao M, Lv L, Ling Z, Li L. The Human Microbiota in Health and Disease. Engineering. 2017;3(1):71–82. doi:10.1016/J.ENG.2017.01.008.

4. Khan R, Petersen FC, Shekhar S. Commensal Bacteria: An Emerging Player in Defense Against Respiratory Pathogens. Frontiers in immunology. 2019;10(1203). doi:10.3389/fimmu.2019.01203.

5. Mazmanian S, Kasper D. The love–hate relationship between bacterial polysaccharides and the host immune system. Nat Rev Immunol. 2006;6:849–858. doi:10.1038/nri1956.

6. Singh S, Verma N, Taneja N. The human gut resistome: Current concepts and future prospects. The Indian journal of medical research. 2019;150(4):345–358. doi:10.4103/ijmr.IJMR_1979_17.

7. Lee K, Raguideau S, Sir/'en K, Asnicar F, Cumbo F, Hildebrand F, et al. Population-level impacts of antibiotic usage on the human gut microbiome. Nature Communications. 2023;14. doi:https://doi.org/10.1038/s41467-023-36633-7.

8. Ghosh TS, Gupta SS, Nair GB, Mande SS. In Silico Analysis of Antibiotic Resistance Genes in the Gut Microflora of Individuals from Diverse Geographies and Age-Groups. PLOS ONE. 2014;8(12):1–15. doi:10.1371/journal.pone.0083823.

9. Robredo B, Singh KV, Baquero F, Murray BE, Torres C. Vancomycin-resistant enterococci isolated from animals and food. Int J Food Microbiol. 2000;54(3):197–204. doi:10.1016/s0168-1605(99)00195-6.

10. Novais C, Coque TM, Costa MJ, Sousa JC, Baquero F, Peixe LV. High occurrence and persistence of antibiotic-resistant enterococci in poultry food samples in Portugal. J Antimicrob Chemother. 2005;56(6):1139–1143. doi:10.1093/jac/dki360.

11. Lupo A, Vogt D, Seiffert SN, Endimiani A, Perreten V. Antibiotic resistance and phylogenetic characterization of Acinetobacter baumannii strains isolated from commercial raw meat in Switzerland. J Food Prot. 2014;77(11):1976–1981. doi:10.4315/0362-028X.JFP-14-073.

12. Dhup V, Kearns AM, Pichon B, Foster HA. First report of identification of livestock-associated MRSA ST9 in retail meat in England. Epidemiol Infect. 2015;143(14):2989–2992. doi:10.1017/S0950268815000126.

13. Buzón-Durán L, Capita R, Alonso-Calleja C. Microbial loads and antibiotic resistance patterns of Staphylococcus aureus in different types of raw poultry-based meat preparations. Poultry Science. 2017;96(11):4046–4052. doi:https://doi.org/10.3382/ps/pex200.

14. Guo H, Pan L, Li L, Lu J, Kwok L, Menghe B, et al. Characterization of Antibiotic Resistance Genes from Lactobacillus Isolated from Traditional Dairy Products. J Food Sci. 2017;82(3):724–730. doi:10.1111/1750-3841.13645.

15. Herreros MA, Sandoval H, González L, Castro JM, Fresno JM, Tornadijo ME. Antimicrobial activity and antibiotic resistance of lactic acid bacteria isolated from Armada cheese (a Spanish goats' milk cheese). Food Microbiology. 2005;22(5):455–459. doi:https://doi.org/10.1016/j.fm.2004.11.007.

16. Mathur S, Singh R. Antibiotic resistance in food lactic acid bacteria–a review. International Journal of Food Microbiology. 2005;105(3):281–295. doi:10.1016/j.ijfoodmicro.2005.03.008.

17. Jahan M, Zhanel GG, Sparling R, Holley RA. Horizontal transfer of antibiotic resistance from Enterococcus faecium of fermented meat origin to clinical isolates of E. faecium and Enterococcus faecalis. International journal of food microbiology. 2015;199:78–85. doi:10.1016/j.ijfoodmicro.2015.01.013.

18. Fraqueza MJ. Antibiotic resistance of lactic acid bacteria isolated from dry-fermented sausages. International journal of food microbiology. 2015;212:76–88. doi:10.1016/j.ijfoodmicro.2015.04.035.

19. Hu X, Zhou Q, Luo Y. Occurrence and source analysis of typical veterinary antibiotics in manure, soil, vegetables and groundwater from organic vegetable bases, northern China. Environmental pollution. 2010;158(9):2992–2998. doi:10.1016/j.envpol.2010.05.023.

20. Marti R, Scott A, Tien YC, Murray R, Sabourin L, Zhang Y, et al. Impact of Manure Fertilization on the Abundance of Antibiotic-Resistant Bacteria and Frequency of Detection of Antibiotic Resistance Genes in Soil and on Vegetables at Harvest. Applied and environmental microbiology. 2013;79(18):5701–5709. doi:10.1128/AEM.01682-13.

21. Schwaiger K, Helmke K, Hölzel CS, Bauer J. Antibiotic resistance in bacteria isolated from vegetables with regards to the marketing stage (farm vs. supermarket). International journal of food microbiology. 2011;148(3):191–196. doi:10.1016/j.ijfoodmicro.2011.06.001.

22. Bakri MM, Brown DJ, Butcher JP, Sutherland AD. Clostridium difficile in ready-to-eat salads, Scotland. Emerging infectious diseases. 2009;15(5):817–818. doi:10.3201/eid1505.081186.

23. Pesavento G, Calonico C, Ducci B, Magnanini A, Lo Nostro A. Prevalence and antibiotic resistance of Enterococcus spp. isolated from retail cheese, ready-to-eat salads, ham, and raw meat. Food Microbiology. 2014;41:1–7. doi:10.1016/j.fm.2014.01.008.

24. Duran GM, Marshall DL. Ready-to-Eat Shrimp as an International Vehicle of Antibiotic-Resistant Bacteria. J Food Prot. 2005;68(11):2395–2401. doi:10.4315/0362-028x-68.11.2395.

25. Shen Y, Liu Y, Zhang Y, Cripe J, Conway W, Meng J, et al. Isolation and Characterization of Listeria monocytogenes Isolates from Ready-To-Eat Foods in Florida. Applied and environmental microbiology. 2006;72(7):5073–5076. doi:10.1128/AEM.00435-06.

26. Goossens H, Ferech M, Vander Stichele R, Elseviers M. Outpatient antibiotic use in Europe and association with resistance: a cross-national database study. Lancet. 2005;365(9459):579–587. doi:10.1016/S0140-6736(05)17907-0.

27. Forslund K, Sunagawa S, Kultima JR, Mende DR, Arumugam M, Typas A, et al. Country-specific antibiotic use practices impact the human gut resistome. Genome Research. 2013;23(7):1163–1169. doi:10.1101/gr.155465.113.

28. Hu Y, Yang X, Qin J, Lu N, Cheng G, Wu N, et al. Metagenome-wide analysis of antibiotic resistance genes in a large cohort of human gut microbiota. Nature Communications. 2013;4. doi:10.1038/ncomms3151.

29. Versporten A, Bolokhovets G, Ghazaryan L, Abilova V, Pyshnik G, Spasojevic T, et al. Antibiotic use in eastern Europe: a cross-national database study in coordination with the WHO Regional Office for Europe. The Lancet. 2014;14(5):381–387. doi:10.1016/S1473-3099(14)70071-4.

30. Lu N, Hu Y, Zhu L, Yang Z, Yin Y, Lei F, et al. DNA microarray analysis reveals that antibiotic resistance-gene diversity in human gut microbiota is age related. Scientific Reports. 2014;4(4302). doi:10.1038/srep04302.

31. Wu L, Xie X, Li Y, Liang T, Zhong H, Ma J, et al. Metagenomics-Based Analysis of the Age-Related Cumulative Effect of Antibiotic Resistance Genes in Gut Microbiota. Antibiotics. 2021;10(8). doi:10.3390/antibiotics10081006.

32. Flores-Mireles AL, Walker JN, Caparon M, Hultgren SJ. Urinary tract infections: epidemiology, mechanisms of infection and treatment options. Nat Rev Microbiol. 2016;13(5):269–284. doi:10.1038/nrmicro3432.

33. Mazzaril A, Bazaj G, Cornaglia G. Multi-drug-resistant Gram-negative bacteria causing urinary tract infections: a review. J Chemotherapy. 2017;29(sup1):2–9. doi:10.1080/1120009X.2017.1380395.

34. Möller TSB, Overgaard M, Nielsen SS, Bortolaia V, Sommer MOA, Guardabassi L, et al. Relation between tetR and tetA expression in tetracycline resistant Escherichia coli. BMC Microbiology. 2016;16. doi:https://doi.org/10.1186/s12866-016-0649-z.

35. Pormohammad A, Nasiri MJ, Azimi T. Prevalence of antibiotic resistance in Escherichia coli strains simultaneously isolated from humans, animals, food, and the environment: a systematic review and meta-analysis. Infection and Drug Resistance. 2019;12:1181–1197. doi:10.2147/IDR.S201324.

36. Sáenz Y, Zarazaga M, Briñas L, Lantero M, Ruiz-Larrea F, Torres C. Antibiotic resistance in Escherichia coli isolates obtained from animals, foods and humans in Spain. 2001;18(4):353–358. doi:10.1016/s0924-8579(01)00422-8.

37. Global Action Plan on antimicrobial resistance. Geneva, Switzerland: World Health Organization; 2015. Available from: `https://www.who.int/antimicrobial-resistance/publications/global-action-plan/en/`.
