# Supplementary material for: A model of antibiotic resistance genes accumulation through lifetime exposure from food intake and antibiotic treatment
Source: PLoS One. 2023 Aug 17;18(8):e0289941. doi: 10.1371/journal.pone.0289941 (PMC10434901; doi:10.1371/journal.pone.0289941)
Supplement: Attachment — Submitted filename: Food_Model_PLoS-response_to_reviewers.docx [file pone.0289941.s005.docx]

PONE-D-23-13150

**Todman  *et al.* Response to Reviewers**

Reviewer #1: This is an interesting manuscript attempts to model resistance gene accumulation after lifetime exposure to antibiotics through food and treatment with antibiotics. This study sheds new light on AMR. My comments are minor in nature. Typographical errors are outlined on the appended manuscript. See appended manuscript.

We thank the reviewer. We have corrected the typographical errors; these are highlighted in the Conclusions sections but not highlighted in references 30, 31 and 33.

Reviewer #2: The manuscript describes the development of a model of ARGs accumulation related to food and antibiotic treatment. ARGs and ARBs spread, and antimicrobial resistances in general, is a very important topic that merits extensive research and the manuscript in itself takes on a very interesting approach. However, there are a few questions that must be addressed before publication:
- the manuscript, although generally well written, as several typos and therefore the authors should make a thorough revision of the manuscript;

We have checked the manuscript for typographical errors and corrected these, including those noted by Reviewer 1. These have all been highlighted in the manuscript

- The authors have results and conclusions in the Introduction (page 2, lines 43 - 46). Please remove;

We have removed that text and replaced with the following (which is also highlighted in the revised manuscript):

“We use the model to identify which of these factors, individually and in combination, are most important in explaining the accumulation of ARGs during an individual's lifetime, and so identify potential mitigations against this aspect of antimicrobial resistance.”

- In the materials and methods, E. coli is used without mentioning it in full before;

We are not clear whether this referee wants us to spell out *Escherichia coli* in full or add a line justifying the use of *E. coli* data. We have done both, as highlighted in the text. The added line is:

“*E. coli* data are used here because this is a standard sentinel organism used for food safety testing; however, our resistance model is intended to me more generic.”

- Why the use of 20% and 50% reductions in the different scenarios? These values should be justified

This is an interesting point. In the paper we have sought to balance analysis of wide ranges of parameter values (i.e. in the sensitivity analyses) with specific scenarios for which we can draw histograms. 20% and 50% are chosen to represent modest and large reductions at a scale where we would expect to see effect (where say 1% is unlikely). We have made this point clear in the final paragraph of the Methods (highlighted).

- Figure 4 is presented after line 128, but the explanation comes further down the text, after explaining that there may be other reduction routes, that are for instance shown in Figure 5. The authors should rewrite lines 129-139 so that it is consistent with the presentation of the figures;

We thank the reviewer. We have moved text around to ensure a more logical flow. It is highlighted in the text.

- On lines 169-170 and 174-176 where the authors describe negligible differences and significant reduction, respectively, was statistical analysis performed? If so, show the results. If not, statistical analysis should be conducted to improve the manuscript and give stronger meaning to these results.

We thank the reviewer for this point. The language we used here was a imprecise. We have changed the word ‘significantly’ with ‘materially’ in the sub-heading and in the description of the sensitivity analysis. With regards the first comparison, we have added text to describe the summary statistics of the distributions obtained which makes clear that the differences between the distributions are negligible.
